# A Multicenter Randomized Controlled Pilot Trial Testing the Efficacy and Safety of Pterygopalatine Fossa Puncture Using One Acupuncture Needle for Moderate-to-Severe Persistent Allergic Rhinitis

**DOI:** 10.1155/2020/2975974

**Published:** 2020-02-20

**Authors:** Lu Zhang, Lei Jiang, Kai Cheng, Jian-Hua Fu, Shen Jian-Wu, Ke-Jian Wang, Yu-juan Song, Xian-zhong Meng, Zhi-Xian Xu, Li-He Chen, Meng-Meng Guo, Li-juan Zhang, Li-li Zhang, Da-Zhuo Shi

**Affiliations:** ^1^Xiyuan Hospital, China Academy of Chinese Medical Sciences, No. 1 Xiyuan Caochang Road, Haidian District, Beijing 100091, China; ^2^Rehabilitation Department, Hannover Medical School, Carl-Neuberg-Strasse 1, Hannover 30625, Germany; ^3^Beijing Dacheng Acupuncture Hospital, No. 39 Yuanda Road, Haidian District, Beijing 100097, China; ^4^Rehabilitation Department, Longhua District Central Hospital, Guanlan Avenue No. 187, Shenzhen 518110, Guangdong, China; ^5^Rehabilitation Department, Pudong New Area People's Hospital Affiliated to Shanghai University of Medicine & Health Sciences, Soth Chuanhuan Rd. 490, Shanghai 210200, China

## Abstract

**Objective:**

To compare the efficacy and safety of pterygopalatine fossa puncture using one acupuncture needle inserted through the temporal fossa (intervention) and Chinese verum acupuncture (VA) in patients with moderate-to-severe persistent allergic rhinitis.

**Methods:**

The patients were randomized to an intervention group receiving pterygopalatine fossa puncture with one acupuncture needle for 4 weeks (once or twice weekly, 4–8 sessions in total, with a second course performed if required) or to a control group receiving individualized VA for 4 weeks (twice weekly, eight sessions in total). Patients were followed up 4 weeks later.

**Results:**

Ninety-six participants were assigned to intervention (*n* = 48) or VA (*n* = 48) or VA (*P* > 0.05 in all cases). Compared with the VA, the time to onset of effect in the intervention group was shorter and the duration of effectiveness was longer. The mean clinical waiting time was significantly shorter in the intervention group than in the control group (6.640 ± 3.035 min and 31.19 ± 10.216 min, respectively). The total number of sessions in the VA group was 384; 7 episodes of subcutaneous bleeding occurred but did not require treatment. The total number of sessions in the intervention group was 185. Two cases of subcutaneous bleeding (one of local hematoma during the intervention and the other one of bruising in the palpebra inferior on the day after intervention) resolved upon withdrawal from the study.

**Conclusions:**

Pterygopalatine fossa puncture using one acupuncture needle resulted in a shorter time to onset of effect, a longer duration of effectiveness, and less clinical waiting time when compared with VA. Though the significant differences for TNSS and TNNSS were shown within intervention and VA groups, there were no differences between the two groups. Although the rate of subcutaneous bleeding was low, these adverse events may influence patient compliance. This trial is registered with ISRCTN21980724.

## 1. Background

Allergic rhinitis is a symptomatic disorder of the nose induced by immunoglobulin E- (IgE-) mediated inflammation of the nasal membranes after allergen exposure [1] and can be classified into intermittent and persistent allergic rhinitis (PAR). Allergic rhinitis has a reported prevalence of approximately 10%–20% globally and 11.1% in China [2]. It causes major illness and disability worldwide and reduces the quality of life and productivity, regardless of ethnicity, gender, or age [3]. Some patients develop asthma, further increasing the associated medical and social burdens.

Conventional therapeutic agents for PAR include H1 antihistamines, glucocorticosteroids, leukotriene antagonists, decongestants, anticholinergics, and specific immunotherapy [4]. However, some patients experience side effects, become dissatisfied, and seek complementary and alternative treatments [5, 6]. In traditional Chinese medicine, physicians have effectively used verum acupuncture (VA) to treat allergic rhinitis for many years [7, 8], and one study has shown the efficacy of acupuncture [9]. Clinical practice guidelines state that clinicians may offer acupuncture to patients with allergic rhinitis who are interested in nonpharmacologic therapy or refer them to a clinician who can offer acupuncture [10]. Interestingly, a large case study suggested that pterygopalatine fossa puncture with one acupuncture needle, a technique developed by a Chinese otolaryngologist and applied in more than 130,000 Chinese patients [11], offers potential advantages with regard to nasal symptoms, time to onset of effect, duration of effectiveness, quality of life, and time required to perform the procedure. However, there has been no randomized controlled trial evaluating the advantage of this novel technique in comparison with VA.

Some patients undergoing the new technique in the clinic developed lower eyelid bruising on the day after the intervention. This adverse reaction cannot be detected at the completion of treatment and may have a negative impact on the health of the patient and may trigger a medical dispute.

We conducted a randomized controlled trial to evaluate the efficacy and safety of this new technique in comparison with VA.

## 2. Methods

### 2.1. Study Design and Ethical Approval

The study was a multicenter, randomized, parallel, VA-controlled, assessor-blinded trial. Three centers in Beijing participated in the study, including Xiyuan Hospital, Beijing Baiwan Chinese Medical Clinic, and Beijing Dacheng Acupuncture Hospital. Beijing Tongren Hospital decided to join the trial at first but withdrew before the trial began because the participating doctor retired. The study design and methodology adhered to the principles of the Declaration of Helsinki and were approved by the Xiyuan Hospital Ethics Committee (December 31, 2013; approval number 2013XL062-2). Full details of the trial protocol can be found online (http://www.controlled-trials.com/ISRCTN21980724, http://www.trialsjournal.com/content/16/1/183).

### 2.2. Inclusion/Exclusion Criteria

To be eligible, participants were required to have been previously diagnosed with moderate-to-severe PAR, according to the Allergic Rhinitis and its Impact on Asthma criteria, and to meet the following requirements: having PAR more than 4 days/week for more than four consecutive weeks, with a disease course of more than 1 year; being aged 18–60 years; completing an allergic rhinitis baseline questionnaire and providing written informed consent; and having a physical sign score ≥1 [12] and a symptom score ≥4 [13].

Patients were excluded if they had any of the following: acute sinusitis, active asthma, or diagnosis or suspicion of pneumonia; nasal abnormalities or rhinopolypus (polypoid lesions were included); a history of taking antihistamines, anticholinergics, corticosteroids, decongestants, or antibiotics in the 2 weeks before enrollment; received systemic corticosteroids within 6 months or specific immune therapy within 1 year before enrollment; received an alternative and complementary modality, i.e., acupuncture or herbal medication, for treatment of PAR within 2 months before enrollment; intended to become or were pregnant; had a serious medical condition, such as uncontrolled hypertension, diabetes mellitus requiring insulin, past or current malignant tumor, severe dyslipidemia, liver or kidney dysfunction, anemia, active pulmonary tuberculosis, an infectious disease, or a systemic disease that cannot be treated by acupuncture; or had a background of heavy smoking.

### 2.3. Sample Size Calculation

The symptomatic outcome of a pilot study (with 10 cases in each group) was applied to calculate the sample size using PASS 2008 software (NCSS, Kaysville, UT, USA). The mean total nasal symptom score (TNSS) [13], assessed serially during the 4-week treatment period, was 4.2071 ± 2.58702 after pterygopalatine fossa puncture with one acupuncture needle and 7.4393 ± 2.0368 after VA. For a power of 0.9 to detect a significant difference (*α* = 0.01, two-sided), 15 participants per group were required. Considering there might be variance between centers, to guarantee the statistical power test, we at last involved 96 patients (48 participants in each group) into the study.

### 2.4. Randomization and Blinding

The central randomization was implemented by Xiyuan Hospital using a block randomization technique to generate the random allocation sequence and to prepare predetermined computer-generated, opaque, sealed randomization envelopes. The envelopes were numbered consecutively and connected to a strain. Each envelope was separated from the strain and opened in sequence only after the run-in period when each participant was registered in the trial. Outcome assessors and personnel who dealt with the data collection and data analysis were blinded throughout the entire trial. The acupuncturist and patients could not be blinded because of the nature of the two different acupuncture techniques; however, the physicians were trained not to communicate with the participants or outcome assessors regarding the treatment procedures and responses. To ensure that all practices at each of the three hospitals were the same, the physicians who enrolled participants and the assessors who collected the data were asked to participate in a 3-day training seminar on treatment modalities and trial documentation prior to the trial. Periodic check-ups on the practices used were performed at each hospital.

### 2.5. Recruitment and Procedure

**Figure 1:**
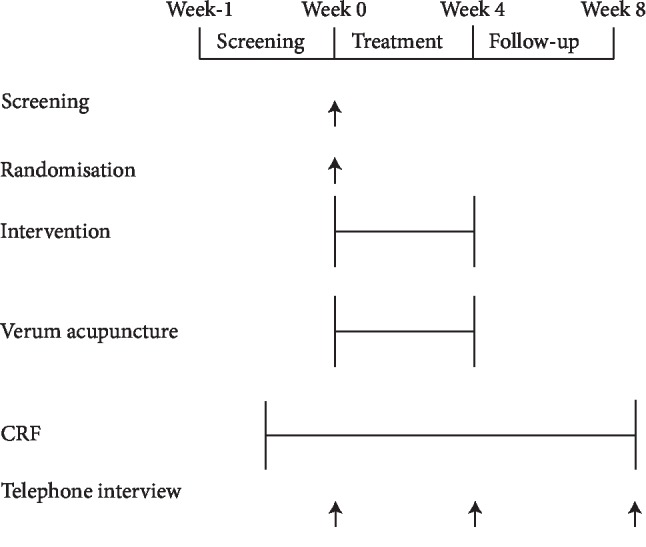
Trial profile.

Patients were recruited from the outpatient clinics of the three participating hospitals between February 4, 2014, and March 21, 2015. Posters describing the required population, offering free blood and allergy testing of eligible patients, and providing the contact information of the researchers were displayed in the clinics. Television advertisements were used to publicize the study. Based on the predetermined randomization envelopes, participants were randomly allocated to the intervention or control group. The patients had an equal probability of being assigned to either of the two groups. Participants were asked to record symptoms in a rhinitis diary from the run-in period to week 4 after randomization. Use of acute symptomatic relief medication was also recorded during the treatment period. The case report form contained all of the outcome measures, and the rhinitis diaries were collected separately from the three hospitals at the end of week 4 after randomization by blinded interviewers. Blinded telephone interviewers contacted the participants regarding the days of moderate-to-severe allergic rhinitis during the 4 weeks since treatment to evaluate the long-term effect of acupuncture at week 8 after randomization (Figure 1).

### 2.6. Intervention

Participants first underwent a standardized interview and received further information regarding the study. Acupuncturists for the control group were required to have over 10 years of clinical experience and an acupuncture license issued by the Ministry of Health of the People's Republic of China. In addition to these criteria, acupuncturists for the interventional group were required to have been trained by Professor Li Xinwu, the inventor of the technique of pterygopalatine fossa puncture with one acupuncture needle; have relevant neuroanatomic knowledge; be able to perform the technique clinically; have practiced no fewer than 10 times under the supervision of Professor Li Xinwu; and have practiced independently no fewer than 2000 times. Before the trial, all acupuncturists received specific training regarding the purpose and procedures of the trial, therapeutic strategies, and quality control.

**Figure 2:**
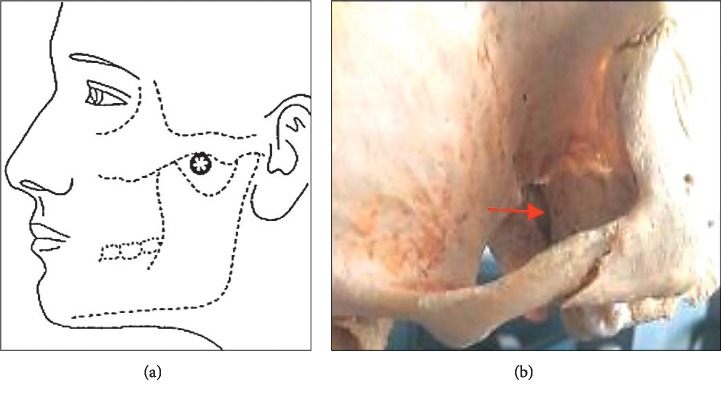
The insertion point and the pterygopalatine fossa. (a) Insertion point for sphenopalatine ganglion stimulation. (b) The red arrow indicates the pterygopalatine fossa in the skull. The figure in (a) is cited from Grégoire et al. [14].

Patients received 4 weeks of treatment. In the interventional group, one disposable sterile acupuncture needle (0.35 mm diameter, 60 mm length; Beijing ZhongyanTaihe Medicine Co., Beijing, China) was inserted into the pterygopalatine fossa. After local disinfection, the needle was gradually inserted between the zygomatic arch and the coronoid process of the mandible to a depth of approximately 55 mm to enter the pterygopalatine fossa (Figure 2). Once the patient experienced a specific sensation (radiating toward the nose), the patient made a hand signal and the needle was then withdrawn immediately. The intervention was applied unilaterally in a session. Patients received 1 or 2 sessions a week, but most required only one weekly session. The physicians decided whether another session was required based on physical signs and symptoms during the second visit of the week.

The control group underwent two sessions of VA per week. The acupoints, including the main and adjunct points (Table 1), were selected on the basis of the Chinese medicine guidelines for allergic rhinitis [15] and were described according to standard nomenclature [16]. Two main and two adjunctive points were applied according to the patient's symptoms (Table 1). In particular, 32-gauge (0.25 mm diameter, 25 mm length) sterile needles (Beijing ZhongyanTaihe Medicine Co.) were used for points on the face, head, and back, whereas 30-gauge (0.3 mm diameter, 40 mm length) sterile needles (Beijing ZhongyanTaihe Medicine Co.) were used for the limb and lumbar points. The depth of insertion ranged between 10 mm and 30 mm (see Table 1). The acupuncturists manually manipulated the acupuncture needles until de-qi (i.e., sensation of tension around the needle felt by the practitioner, and numbness, distension, soreness, and heaviness around the acupoint felt by the patient) and maintained the needle position for 25 min.

### 2.7. Baseline Assessment

During the run-in period (baseline), the physical sign score (measured at least twice), the TNSS [13], and total nonnasal symptom score (TNNSS) [17] measured once daily, and Rhinoconjunctivitis Quality of Life Questionnaire (RQLQ) score [18, 19] (measured at least once) were determined.

### 2.8. Outcome Measures

The primary outcome measure was the change in TNSS between baseline (week 0) and week 4. Secondary outcome measures included change in TNNSS and RQLQ scores from baseline to week 4, change in number of symptomatic days from baseline to the end of the follow-up period (week 8), time to onset of effect and duration of effectiveness at every session, clinical time spent (defined as waiting time in hospital plus treatment time during each session), change in total IgE level, and eosinophil count in venous blood from baseline to week 4 (Table 2). Time to onset of effect was the timing of changes in the physical sign score and degree of nasal congestion. The physical sign score was calculated from the degree of swelling of the inferior nasal concha [12] before and after each session. The degree of nasal congestion was scored using a visual analog scale [20] before and after each session. The duration of effectiveness was recorded as the duration of change in TNSS after each session (once the patient experienced alleviation of symptoms).

### 2.9. Statistical Analysis

Data were analyzed by intention-to-treat analysis (participants who had at least one measurable outcome after treatment; missing data were replaced according to the principle of last observation carried forward). A variance analysis was performed to reject the global hypothesis that “there is no difference in all fields of means between the groups.” The significance level was set at 5%, with *P* < 0.05 indicating a significant difference.

Mean TNSS and TNNSS were compared by analysis of variance for repeated measures between the groups. Mean time to onset of effect and duration of effectiveness was compared between the groups by the *t*-test. Analysis of variance for repeated measures was used for between-group comparisons of RQLQ scores. The other continuous variables (total IgE level and eosinophil count in venous blood, number of symptomatic days, and clinical time spent) were compared by the *t*-test. Comparisons within groups were performed by multivariate analysis of variance followed by Tukey's post hoc test. Within the three hospitals, stratified analysis was performed to control for confounding factors if necessary. The incidence of adverse events was calculated and compared between the groups using the chi-square test or Fisher's exact test. All analyses were performed using Statistical Package for the Social Sciences version 13.0 software (SPSS, Chicago, IL, USA).

## 3. Results

### 3.1. Demographic Data

Of the 138 patients screened, 96 participants were randomly allocated to the intervention group or VA group (*n* = 48 in each). The subjects were recruited between February 4, 2014 and March 21, 2015, and the trial ended on May 23, 2015.

**Figure 3:**
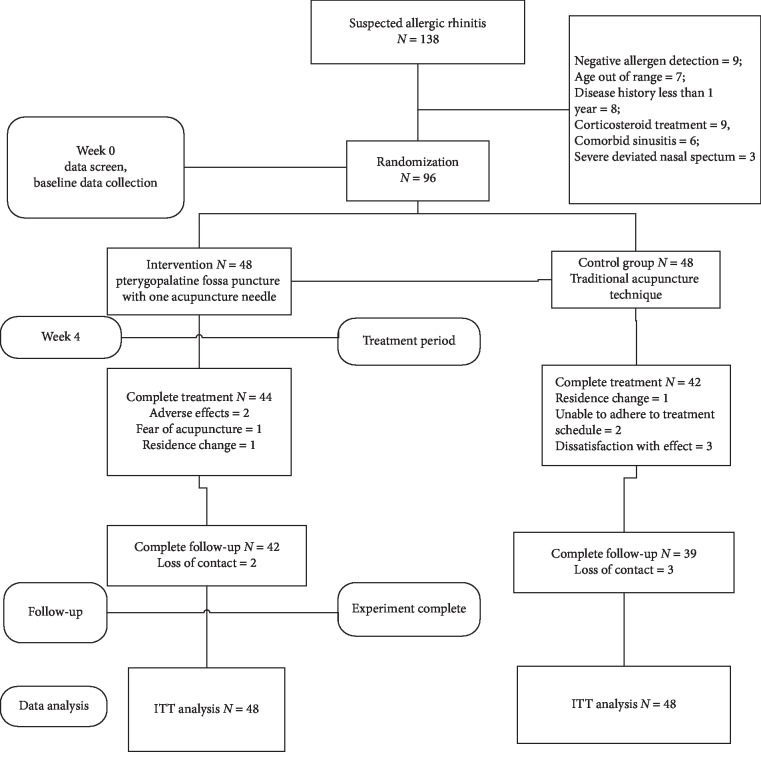
Flow profile for this clinical trial.

Fifteen patients dropped out of the study, with a rate of 12.5% (6/48) in the intervention group and 18.8% (9/48) in the control group. The reasons included time restrictions, change in residence, dissatisfaction, fear of needling, and change in contact information during the follow-up period; the details are shown in Figure 3.

No significant differences were identified between the subjects in the two groups with regard to age, gender, disease course, number of symptomatic days in the previous month, TNSS, TNNSS, RQLQ score, physical sign score, and baseline parameters in the intention-to-treat population (Table 3).

### 3.2. Primary Outcome

After the first week, there were significant reductions in TNSS in both groups; the differences were −2.79 (95% confidence interval [CI] −3.92, −1.65, *P* < 0.001) for the intervention group and −3.05 (95% CI −4.01, −2.08, *P* < 0.001) for the control group. The significance increased up to the end of the treatment period; differences at the fourth week after randomization were −5.24 (95% CI −7.17, −3.69, *P* < 0.001) for the intervention group and −4.89 (95% CI −6.10, −3.68, *P* < 0.001) for the control group (Table 3).

However, the difference in TNSS between the two groups was not significant during the treatment period; the respective F and *P* values were 1.54 and 0.22 in the first week, 0.30 and 0.58 in the second week, 0.02 and 0.88 in the third week, and 0.03 and 0.86 in the fourth week.

### 3.3. Secondary Outcomes

After the first week, compared with baseline, there were significant reductions in the TNNSS and Nasal Symptom Score 2004 Chinese version in each group; the differences were −0.58 (95% CI −1.01, −0.14, *P* < 0.05) and −1.37 (95% CI −2.11, −0.62, *P* < 0.01) for the intervention group and −0.46 (95% CI −0.91, −0.01, *P* < 0.05) and −1.37 (95% CI −2.02, −0.72, *P* < 0.01) for the control group. The significance continued up to the end of the treatment period; differences at week 4 after randomization were −0.87 (95% CI −1.46, −0.29, *P* < 0.01) and −2.77 (95% CI −3.89, −1.64, *P* < 0.01) for the intervention group and −1.25 (95% CI −1.01, −0.14, *P* < 0.05) and −2.68 (95% CI −3.58, −1.788, *P* < 0.01) for the control group (Table 2). However, the differences in TNNSS and Nasal Symptom Score 2004 Chinese version between the two groups were not significant during the treatment period; for TNNSS, the respective F and *P* values were 1.27 and 0.26 in the first week, 0.76 and 0.39 in the second week, 0.23 and 0.63 in the third week, and 0.10 and 0.75 in the fourth week. For the Nasal Symptom Score 2004 Chinese version, the respective F and *P* values were 1.41 and 0.24 in the first week, 0.02 and 0.89 in the second week, 0.91 and 0.35 in the third week, and 0.84 and 0.36 in the fourth week.

The RQLQ score did not change significantly between the intervention and VA groups during the treatment period (Table 2). Among the seven domains of the RQLQ, only the sleep and practical problem domains demonstrated significant differences between the intervention and VA groups (*P*=0.035 at 3 weeks for the sleep domain, *P*=0.023 at 1 week for the practical problem domain; Table 4). However, after the first week, a significant reduction in RQLQ was observed in each group when compared with baseline; the differences were −19.37 (95% CI −25.54, −13.19, *P* < 0.001) for the intervention group and −16.03 (95% CI −22.47, −9.59, *P* < 0.001) for the control group. The significance increased up to the end of the treatment period; the differences at week 4 after randomization were −41.13 (95% CI −50.47, −31.79, *P* < 0.001) for the intervention group and −28.98 (95% CI −36.68, −21.25, *P* < 0.001) for the control group (Table 4).

There were fewer symptomatic days in both groups during the follow-up period when compared with baseline (*P* < 0.05); however, there was no significant difference in this regard between the two groups (*P* < 0.05).

There was a significantly shorter time to onset of effect and a significantly longer duration of effectiveness in the intervention group (both *P* < 0.001). The mean clinical waiting time was significantly shorter in the intervention group than in the control group (6.640 ± 3.035 min and 31.19 ± 10.216 min, respectively; *P* < 0.01).

There were no statistically significant differences in venous blood eosinophil counts or and in total IgE levels between baseline and after treatment in either group or between baseline and after treatment between the two groups (both *P* > 0.05).

### 3.4. Safety

One patient in the intervention group was withdrawn from the study due to bruising of the lower eyelid that appeared on the day following treatment. The patient received oral cephalosporin treatment for 3 days to prevent secondary infection, and the eyelid bruises disappeared 1 day after the start of antibiotic treatment. In the control group, there were six cases of subcutaneous bruising, all of which disappeared on the following day with no special treatment.

## 4. Discussion

Traditional acupuncture technique is popular in China and was recommended by China Association of Acupuncture and Moxibustion. Acupuncture can produce a therapeutic effect on allergic rhinitis by restoring Th1/Th balance, decreasing serum immunoglobulin E levels, reducing nasal mucosal inflammatory cell infiltration, and regulating substance *P* levels [21]. However, a modern acupuncture technique, pterygopalatine fossa puncture with one acupuncture needle, is not only effective in treating allergic rhinitis but also better in the field of rapid onset of effect and longer duration of therapeutic efficacy compared with a traditional acupuncture technique, so that it might be more advantageous for treating moderate-to-severe PAR. However, no experiment has been performed previously to test this possibility. This is the first multicenter randomized controlled trial to validate the difference in efficacy between a new and a traditional acupuncture technique and to assess the safety of this new technique. The baseline parameters in this study, including age, gender, disease course, number of symptomatic days in the previous month, and scale scores for the TNSS (2004 Chinese version), TNNSS, and RQLQ were not significantly different between the intervention and control groups.

Of the 96 patients recruited, 15 (15.63%) withdrew from the study; this is lower than the dropout rate limit of 20% in clinical studies, indicating an acceptable level of patient compliance. In addition to three cases involving a change in residence and job-related issues, three cases in the intervention group withdrew from the study due to adverse effects and a fear of acupuncture and three cases in the control group withdrew due to dissatisfaction with therapeutic efficacy, all of which were related to the treatment strategies themselves. In addition, two and three cases in the intervention group and control group, respectively, were lost to follow-up, which is not unusual in clinical studies.

The primary outcome, TNSS, and the secondary outcomes, including the TNNSS (2004 version), RQLQ score, and number of symptomatic days during follow-up, were not significantly different between the two groups (all *P* > 0.05). The average scores for the above parameters at the different time points were significantly different from the baseline scores in both groups (all *P* < 0.01), indicating that both the new intervention and the traditional acupuncture technique were effective for moderate-to-severe PAR and reduced the number of symptomatic days during follow-up. However, there was no significant difference in efficacy between the two treatments. The change in total IgE level and eosinophil count in venous blood from baseline to week 4 did not show statistically significant differences between or within groups, indicating that the two acupuncture techniques did not affect the two biomarkers associated with allergic rhinitis.

This study evaluated the time to onset of effect, duration of therapeutic efficacy, and clinical waiting time in the intervention and control groups, and the results indicated that the differences in time to onset of effect and duration of therapeutic efficacy between the two treatments were statistically significant (all *P* < 0.001). The time to onset of effect in the intervention group was shorter than that in the control group, and the duration of therapeutic efficacy was longer in the intervention group than in the control group, indicating that the new acupuncture technique was more effective in terms of the above clinical outcomes than the traditional acupuncture technique. The clinical waiting time in the intervention group was shorter than that in the control group (*P* < 0.001), which indicates that this intervention consumes less time from the patient's perspective and in turn results in better cost-effectiveness.

In the intention-to-treat analysis, of a total of 336 sessions in the control group, mild subcutaneous bleeding was observed on only seven occasions, and did not warrant withdrawal from the study in any patient. In the intervention group, of a total of 189 sessions, subcutaneous bleeding was observed twice (local hematoma was observed once in the course of a session, and lower eyelid bruising on the treated side was observed on the day after a session). The local hematoma and lower eyelid bruises disappeared after cessation of treatment and did not subsequently impact the health of the patients concerned. Clinically, treatment could have been continued in these two patients if they had not chosen to withdraw from the study. Therefore, pterygopalatine fossa puncture with one acupuncture needle technique and traditional acupuncture are both safe techniques, but the former may affect the compliance of some patients due to side effects.

The anatomical literature suggests that the maxillary nerve and pterygopalatine segment of the maxillary artery are located in the pterygopalatine fossa and that the needle may puncture the artery or its branches and cause lower eyelid bruising. To test this hypothesis, we conducted an anatomical study to simulate the path of needle insertion in the clinical intervention procedure. The findings suggested that the lower eyelid bruising on the treated side occurring on the day after the acupuncture session was due to “the needle puncture of the pterygopalatine segment of the maxillary artery or its anterior branching infraorbital artery.”

The intervention described in the protocol involves treating the sphenopalatine ganglion with one acupuncture needle. In order to test whether the sphenopalatine ganglion can be touched by the acupuncture needle, to determine if this method can reduce the incidence of adverse effects, and to improve the acupuncture technique, we conducted an anatomical study using six adult wet male skulls in which we measured the depth of needle insertion and the distance between the needle and the sphenopalatine ganglion. We found that by passing through the lower temporal fossa, the needle can enter into the bony anatomical structure and the pterygopalatine fossa, in which the sphenopalatine ganglion is located. However, of the 12 acupuncture procedures, the needle came into direct contact with the sphenopalatine ganglion in only two procedures, indicating that in most cases, the needle only entered into the pterygopalatine fossa. The researchers believe there is a lack of reliable evidence to support the idea that direct contact between the sphenopalatine ganglion and acupuncture needles accounts for all the clinical effects seen using acupuncture in PAR. Clinical observations suggest that when the acupuncture needle reaches a certain depth, nasal symptoms and physical signs will be improved. With reference to other nerve stimulation techniques [22] and consultations with experts in neurology, the researchers believe that acupuncture may produce therapeutic effects as long as the needle is in close proximity to the nerves. Therefore, the probability of the needle being in close proximity to the sphenopalatine ganglion (defined as a distance between the needle and the ganglion of less than 10 mm) was measured, and the results revealed that the needle approached the sphenopalatine ganglion in 8 of 12 procedures. Thus, the researchers propose that the expression “pterygopalatine fossa puncture with one acupuncture needle” is more accurate and should be recommended to replace the phrase “sphenopalatine ganglion with one acupuncture needle” [23], and the first term about the intervention was described in 1990 in the case report: “the use of acupuncture at the sphenopalatine acupoint to treat allergic rhinitis.”

Some of the patients in the intervention group who need only one session per week can gain the satisfied efficacy, and in order to reduce the side effect rate of the new acupuncture technique, the doctor will interview the patient review and the clinical symptoms and check the patient's inferior nasal concha swelling situation and then decide whether the patient needs the second session or not.

The main limitation of this study is that, based on ethical principles, participants were allowed to take medication for acute symptomatic relief (i.e., cetirizine hydrochloride 10 mg) if they experienced intolerable symptoms that developed at home. In total, 15 tablets were taken by three patients in the control group and one in the intervention group. Although the amount was small, this may have introduced bias. In addition, the sample size was relatively small.

## 5. Conclusion

A more precise expression for the “sphenopalatine ganglion acupuncture technique” should be “pterygopalatine fossa puncture technique with one acupuncture needle.” Though the significant differences for TNSS and TNNSS were shown within intervention and VA groups, there were no differences between the two groups. The advantages of the new technique over the traditional one include rapid onset, long duration of effectiveness, and shorter treatment and waiting times. Further studies are warranted to reduce the incidence of adverse effects caused by the novel technique.

## Figures and Tables

**Table 1:** Verum acupuncture points and techniques for moderate-to-severe persistent allergic rhinitis.

Acupoint	Direction	Depth (mm)
*Main*		
Yingxiang (LI20), both	Transversely along the nasolabial sulcus and toward the root of the nose	10–15
Yintang (GV29)	Transversely and downward toward the nose	10–15
Fengchi (GB20)	Obliquely toward the tip of the nose	13–20
Fengfu (GB16)	Perpendicular to the skin	13–20
Zusanli (ST36)	Perpendicular to the skin between the tibia and the fibula	20–30

*Adjunct*		
Shangxing (GV23)	Transversely toward the calvarium	8–13
Hegu (LI4)	Perpendicular to the skin	13–25
Kouheliao (LI19)	Obliquely toward the tip of the nose	5–8
Feishu (BL13)	Obliquely toward the spine	13–20
Pishu (BL20)	Obliquely toward the spine	13–20
Shenshu (BL23)	Perpendicular to the skin	15–25
Sanyinjiao (SP36)	Perpendicular to the skin	13–25

**Table 2:** Changes in the TNSS, TNNSS, 2004 version, RQLQ scores, and other secondary outcomes.

	Intervention (*n* = 48)	Verum acupuncture (*n* = 48)	*P* value
*TNSS*			
1 week	7.87 ± 2.47	6.81 ± 3.53	0.223
2 weeks	6.68 ± 3.02	6.02 ± 3.03	0.579
3 weeks	5.51 ± 3.33	5.41 ± 3.01	0.884
4 weeks	5.32 ± 3.91	5.01 ± 3.23	0.861

*TNNSS*			
1 week	2.34 ± 1.23	2.31 ± 1.41	0.971
2 weeks	2.32 ± 1.33	2.21 ± 1.23	0.861
3 weeks	2.12 ± 1.33	1.99 ± 1.12	0.589
4 weeks	2.01 ± 1.41	1.61 ± 1.21	0.253

*2004 version*			
1 week	7.12 ± 1.91	6.51 ± 2.13	0.281
2 weeks	6.41 ± 2.19	6.32 ± 2.32	0.913
3 weeks	6.12 ± 2.14	5.63 ± 2.42	0.451
4 weeks	5.72 ± 2.61	5.24 ± 2.67	0.482

*RQLQ*			
1 week	59.34 ± 18.21	56.92 ± 25.31	0.721
2 weeks	48.91 ± 20.23	53.21 ± 26.37	0.502
3 weeks	40.42 ± 22.67	48.98 ± 24.57	0.185
4 weeks	37.54 ± 26.15	44.13 ± 25.37	0.321
Onset time	465.000 (rank sum)	1365.000 (rank sum)	<0.001
Duration of effectiveness	77.67 ± 31.25 (hour)	48.15 ± 13.31 (hour)	<0.001
Clinical waiting time	6.64 ± 3.04 (min)	31.19 ± 10.23 (min)	<0.001
Eosinophil count	0.29 ± 0.181	0.27 ± 0.172	0.304
Immunoglobulin E	232.49 ± 171.23	191.84 ± 167.37	0.84

^*∗∗*^ *P* < 0.01, ^*∗∗∗*^*P* < 0.001. TNSS, total nasal symptom score; TNNSS, total nonnasal symptom score; RQLQ, Rhinitis Quality of Life Questionnaire.

**Table 3:** Demographic data for the participants in each group.

	Intervention (*n* = 48)	Verum acupuncture (*n* = 48)	*P* value	Statistic
Age (years)	39.03 ± 10.25	44.73 ± 9.72	0.456	*t*-test
Gender (male/female)	15/33	20/28	0.417	Chi-squared test
Disease course (months)	52.47 ± 49.12	58.12 ± 64.47	0.846	Mann–Whitney test
Symptomatic days in the last month	25.87 ± 5.79	23.61 ± 8.34	0.403	Mann–Whitney test
TNSS	11.47 ± 2.35	9.92 ± 2.67	0.278	Multivariate ANOVA
TNNSS	2.81 ± 1.12	2.72 ± 1.51	0.761	Multivariate ANOVA

RQLQ	80.11 ± 18.12	74.82 ± 24.93	0.801	Multivariate ANOVA
Activities	1214 ± 3.32	12.51 ± 5.91	0.683	
Sleep	6.73 ± 4.31	7.83 ± 4.62	0.273	
Nonnasal/eye	15.81 ± 6.71	16.63 ± 7.82	0.671	
Practical problems	13.93 ± 3.45	13.08 ± 4.21	0.242	
Nasal problems	13.91 ± 2.63	13.72 ± 3.63	0.758	
Eye symptoms	9.37 ± 6.54	7.51 ± 5.07	0.216	
Emotional function	9.33 ± 5.31	7.57 ± 4.87	0.076	

Physical sign (score)	2.23 ± 0.62	2.24 ± 0.61	0.601	Mann–Whitney test

ANOVA, analysis of variance; TNSS, total nasal symptom score; TNNSS, total nonnasal symptom score; RQLQ, Rhinitis Quality of Life Questionnaire.

**Table 4:** Effect of treatment on Rhinitis Quality of Life Questionnaire score.

	Intervention	Verum acupuncture	*P* value
	ITT	ITT	
*Activities*			
Baseline	12.07 ± 3.34	11.53 ± 5.67	0.671
1 week	9.61 ± 3.84	8.93 ± 5.34	0.528
2 weeks	8.72 ± 4.23	7.73 ± 4.73	0.383
3 weeks	7.32 ± 4.21	6.93 ± 4.47	0.672
4 weeks	7.81 ± 4.35	6.78 ± 4.47	0.489

*Sleep*			
Baseline	6.83 ± 4.37	7.82 ± 4.63	0.272
1 week	4.93 ± 2.98	6.28 ± 4.63	0.132
2 weeks	3.93 ± 2.97	5.22 ± 4.21	0.123
3 weeks	3.34 ± 2.87	4.98 ± 3.57	0.033∗
4 weeks	3.15 ± 3.36	4.43 ± 4.01	0.127

*Nonnasal/eye*			
Baseline	15.87 ± 6.73	16.47 ± 7.81	0.672
1 week	13.67 ± 5.54	13.78 ± 7.37	0.924
2 weeks	11.37 ± 6.67	12.87 ± 7.47	0.362
3 weeks	8.81 ± 6.37	10.67 ± 6.34	0.223
4 weeks	7.97 ± 6.32	9.53 ± 6.16	0.283

*Practical problems*			
Baseline	13.23 ± 3.45	12.23 ± 4.27	0.242
1 week	10.67 ± 2.82	9.98 ± 3.34	0.127
2 weeks	8.67 ± 3.32	8.67 ± 3.43	0.947
3 weeks	8.27 ± 3.34	7.89 ± 3.61	0.463
4 weeks	6.87 ± 3.19	7.85 ± 3.25	0.329

*Nasal problems*			
Baseline	12.57 ± 2.27	12.38 ± 3.47	0.747
1 week	9.89 ± 2.87	9.36 ± 3.73	0.531
2 weeks	8.56 ± 3.12	9.21 ± 3.63	0.446
3 weeks	7.22 ± 3.03	8.47 ± 3.69	0.113
4 weeks	6.78 ± 3.46	7.92 ± 3.60	0.121

*Eye symptoms*			
Baseline	9.58 ± 6.59	7.86 ± 4.51	0.029
1 week	5.78 ± 4.67	4.27 ± 3.58	0.084
2 weeks	4.21 ± 3.21	3.78 ± 3.37	0.534
3 weeks	3.39 ± 2.87	3.17 ± 2.91	0.887
4 weeks	2.98 ± 1.87	3.03 ± 2.88	0.936

*Emotional function*			
Baseline	9.83 ± 5.37	7.87 ± 4.98	0.078
1 week	6.47 ± 3.96	6.13 ± 4.27	0.728
2 weeks	4.47 ± 3.43	5.31 ± 4.75	0.315
3 weeks	3.57 ± 3.21	4.25 ± 3.97	0.457
4 weeks	3.25 ± 3.36	3.38 ± 3.91	0.487

^*∗*^ *P* < 0.05. ITT, intention-to-treat.

## Data Availability

The data used to support the findings of this study are available from the corresponding author upon request.

## Abbreviations

PAR: Persistent allergic rhinitis
RQLQ: Rhinoconjunctivitis Quality of Life Questionnaire
TNNSS: Total nonnasal symptom score
TNSS: Total nasal symptom score
VA: Verum acupuncture.
